# Evaluation of Y-site compatibility of home total parenteral nutrition and intravenous loop diuretics

**DOI:** 10.1097/MD.0000000000015747

**Published:** 2019-05-24

**Authors:** Dorota Watrobska-Swietlikowska, Magdalena Pietka, Stanislaw Klek

**Affiliations:** a Department of Pharmaceutical Technology, Medical University of Gdansk, Gdansk; b General Surgery and Oncology Unit, Stanley Dudrick's Memorial Hospital, Skawina, Poland.

**Keywords:** chronic kidney disease, furosemide, oily globule size distribution, parenteral admixture, physicochemical analysis, torasemide

## Abstract

In chronic kidney disease (CKD), the design of the parenteral nutrition (PN) regimen becomes more challenging where only individualized PN is appropriate, coupled with the increased risk of unintended interactions with diuretic therapy. In an effort to ensure safe therapy in the home, we assessed the physical stability of bespoke PN formulations intended for use in CKD in the simultaneous presence of Y-site compatibility of furosemide and torasemide. The patient's daily needs were determined based on both metabolic demands as well as the demand for fluids.

Complete admixtures were subjected to physical stability analysis consisting of visual inspection, a validated light microscope method, pH measurement, zeta potential measurement, and characterization of oily globule size distribution. Y-site compatibility of furosemide and torasemide with the formulated admixtures was also performed.

The total parenteral admixture was stable over 7 days at +4°C and 24 h at +25°C and compatible via the Y-line together with furosemide and torasemide over 12 h at +25°C.

The stability assessment guarantees the safety and efficiency of home PN with loop diuretics therapy in CKD patients. This means that these patients do not need long hospitalization and they can be safely treated at home. Furthermore, this study proved that torasemide is the same safety diuretic as furosemide, which has a great impact on clinical practice.

## 1 Introduction

Home parenteral nutrition (HPN) in patients with chronic kidney disease (CKD) is a huge challenge for nutritional support because it is complicated.^[1]^ The requirements for such patients are quite different from the standard composition of multichamber bags (MCB) and customized formulas that are typically indicated for them. In the clinical practice of our hospital, there was a need to create new stable parenteral formulations with a special composition to reduce the progression of renal insufficiency through the reduction of proteinuria and by the correction of metabolic differences.^[2]^ The modalities of nutritional treatment have been focused on protein and calorific intake, electrolyte balance, and demand for fluids. The composition of new admixtures was proposed. The calculated amount of amino acid was 0.6 g per kg of body weight, the daily energy intake was 20 nonprotein kcal per kg, the electrolyte intake could be individually determined in terms of 0 to 3 mmol per kg of sodium and potassium, 0 to 0.3 mmol per kg of magnesium and calcium, and 0 to 0.9 mmol per kg of phosphate. Volume intake was estimated as 60 mL per kg because of diuretic therapy (Table 1). If a patient receives a continuous infusion of parenteral nutrition (PN), it might be beneficial to coadminister loop diuretics and PN. In practice, 2 methods are used to administer drugs with PN admixtures: simultaneous Y-site infusion or inclusion in the PN admixture. The first method consists of an intermittent infusion of drugs with parenteral nutrition admixture in simultaneous Y-site administration. The PN admixture is used as a vehicle for introducing the drugs into the patient. In this method, the contact time between drugs and parenteral admixture can range from 10 min to 12 h. In the second method, the drugs are mixed together with the PN admixture. The period of co-infusion in the second method is the same as the Y-site administration, usually up to 24 h. However, this method requires the physicochemical stability of PN admixtures in the presence of drugs as well as chemical stability data. The second method is not usual in daily clinical practice for these reasons. PN admixtures are complex formulations with limited stability; therefore, stability tests are required in order to ensure the efficacy and safety of HPN with an extended shelf life. Lipid emulsions are the most unstable components of PN admixtures due to the risk of obstructing pulmonary arterioles as well as emboli.^[3]^ Before adding any drug to PN admixtures or even delivering it by simultaneous Y-site infusion, its physicochemical stability must be reviewed in order to maintain the stability of the PN admixture, especially lipid emulsion (avoiding emulsion breaking, creaming, or drug precipitation) and in order to obtain safe therapy. In clinical practice, pharmacists are frequently consulted about the administration of drugs via PN admixtures but there is a lack of information about the compatibility of some drugs due to the high variability in PN admixture composition. There are many articles about the stability of furosemide with PN admixtures but nothing about using torasemide with PN admixtures. However, there are many studies on furosemide in literature. In our hospital, we cannot guarantee identical working conditions, so we need compatibility studies with PN admixtures dedicated to our patients.

**Table 1:**
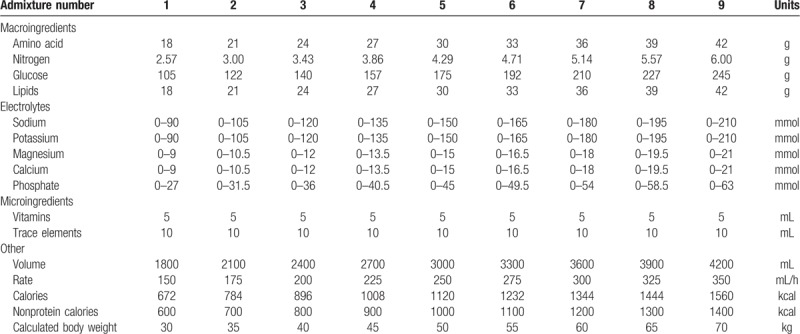
Nutritional requirements for home parenteral nutrition patients with chronic kidney disease per body weight.

The purpose of this work was to obtain compatibility data for 2 loop diuretics—furosemide and torasemide as well as PN admixtures administered to home CKD patients. We created a new stable parenteral formulation with a special composition to reduce renal failure, taking into account metabolic differences in patients. The study used increasing drug concentrations up to the maximum, thus mimicking the range of concentrations and infusion rates that might be relevant for Y-site administration, up to 24 h. This is an approach to extend the experimental area, which is especially important in the clinical practice for HPN therapy in CKD patients. The focus of this study was to assess the physical compatibility of loop diuretics and parenteral admixtures intended for CKD patients.

## 2 Materials and methods

### 2.1 Preparation of parenteral admixtures

All new admixtures were prepared, at the Hospital Pharmacy Unit under aseptic conditions using the automated admixing device Baxa EM2400 (Baxa Corporation, Englewood), supported by the PN calculator Abacus version 3.1. The following commercially available sterile and apyrogenic solutions were used: Aminomel Nephro 6% (Baxter, Poland), glucose 50% (Baxter Healthcare Ltd.), ClinOleic 20% (Baxter), water for injection (Baxter), sodium chloride 10% (B. Braun Melsungen AG, Germany), potassium chloride 15% (Fresenius Kabi AB, Sweden), magnesium sulfate 20% (Polpharma, Poland), calcium chlorate 10% (WZF, Poland), Glycophos (organic phosphate containing solution of sodium glycerophosphate, Fresenius Kabi AB), Nutryelt (trace elements, Baxter), and Cernevit (vitamins, Baxter). All components were mixed as all-in-one admixtures (Table 2) into the final delivery container (Baxa multilayer bag). Admixtures were prepared in 2 different series; in each, 2 bags were collected and transported under a controlled temperature of 2°C to 8°C for subsequent analysis. Diuretics: Furosemide Kabi Injection (10 mg/mL, Fresenius Kabi, Germany) and Trifas 20 Injection (5 mg/mL, Berlin-Chemie, Germany) solutions had been prepared as an infusion just before analysis. Infusion stability was demonstrated for 24 h at room temperature in plastic containers with 100 mL 0.9% sodium chloride (B. Braun) in various concentrations (Table 3).

**Table 2:**
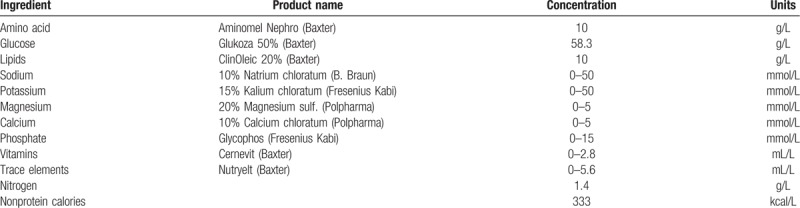
Regimens contents of parenteral nutrition admixture.

**Table 3:**
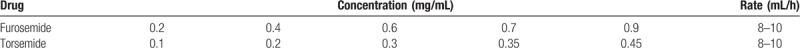
Concentration of the drug (mg/mL) diluted with 0.9% NaCl before adding to parenteral nutrition (mg/mL) and flow rate (mL/h).

### 2.2 Physical analysis of complete PN admixtures and compatibility of diuretics drugs

Samples were subjected to an established panel of methods and acceptance criteria to assess physical incompatibility in terms of potential precipitation or emulsion destabilization. Samples were tested within 5 min after mixing and again after 30 min, 1 h, 2 h, and 4 h. The first test point is referred to as “5 min.” As an additional check, analyses were also performed after 24 h. Samples with drugs were compared with the control—pure PN admixture stored up to 8 days.

The physical stability of parenteral admixtures was assessed by visual inspection: a lipid droplet measured in a light biologic microscope with a camera (B1 223A Motic, Wetzlar, Germany) with an upper droplet size of ≥1 μm. Each microscopic sample (10 μL by a manual pipette) was analyzed with 40-fold magnification. Five individual visual fields were inspected per microscopic sample (15 total visual fields/aliquot): 4 in the corner and 1 in the middle of the preparation. The size of the lipid droplets in the visual field was determined using an ocular micrometer (0.01 mm). The diameter of the largest lipid droplet was measured and counted in each of the 15 visual fields tested per aliquot.

The droplet size of emulsions was determined using photon correlation spectroscopy (PCS), which covers a size range of 20 to 5000 nm (Zetasizer Nano ZS, model ZEN 3600, Malvern Instruments, Malvern, UK). Each sample was determined in triplicate at 25°C. Data are shown in terms of effective mean diameter (Z-average) and the polydispersity index, which reflects the width of the particle size distribution. The second technique was laser diffractometry (MasterSizer E, Malvern Instruments). All results were calculated according to the Mie theory. The dispersions were characterized by their volume diameters D50, D90, and Dmax which means 50% and 90% or all of the particles are below the given size. The charge on the emulsion droplets was measured at 25°C using the moving boundary electrophoresis technique (Zetasizer Nano ZS, Malvern Instruments). Electrophoretic mobility was converted into zeta potential using the Helmholtz–Smoluchowski equation. Twelve readings were recorded for each sample. The pH values of all parenteral admixtures were determined at 25°C (pH meter type 350, Orion-Research, Boston, MA) with a combined electrode (Type ERH-11, Hydromet, Warsaw, Poland). Before each pH measurement, a 2-point calibration of the pH meter was performed, each with a buffer solution of pH 9.00 and pH 4.00, respectively. The pH 7.00 solution was used afterwards as a control. Between the calibration steps, the electrode was rinsed with distilled water and wiped dry. Each sample was measured after 5 min of equilibration.

All PN admixtures were inspected in the presence of oily droplets and coalescence in the presence of furosemide as well as torasemide.

To simulate the mixing in the Y-site (dynamic method), different infusion rates of PN (Table 1) and the drugs (Table 3) were calculated based on the clinical experience and finally, the Y-site administration was realized. In static methods, the mixing ratios of PN and the drugs in the infusion line were calculated and finally 180 mL of total parenteral nutrition (TPN) admixture and 11 mL of drug solution was mixed in 1 container. After 5 min, 30 min, 1 h, 2 h, 4 h, and 24 h samples were taken for testing. All analyses were referred to the parenteral admixture without any drugs.

### 2.3 Statistical analysis

The results are presented as mean ± standard deviation values. All measurements were made in triplicate. For multiple comparisons, the ANOVA test was used. The priori level of significance was 0.05.

## 3 Results

Critical parameters relating to the stability of parenteral admixtures, including the distribution of oily droplets, Z-average, pH, and zeta potential were examined in the presence of loop diuretics (Table 4). The composition of admixtures is presented in Table 2.

**Table 4:**
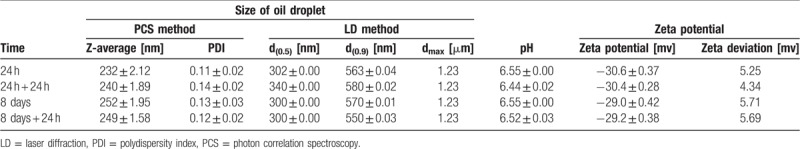
Physical characteristic of pure parenteral admixture (without drugs).

Over 24 h of contact reaction, no visual changes were observed in the test samples stored at room temperature. There was no creaming or discoloration. There was no visible evidence of precipitation or flocculation. A visual inspection was carried out for the assessment of large particle formation in the critical size of 1 to 5 μm to avoid embolism.

Under microscopic observation, despite the presence of furosemide as well as torasemide, the mean of the largest lipid droplet in μm out of 15 visual fields of 5 μm as the upper limit value for the emulsion stability was never reached by any sample over all analyses periods. The mean value of the larger oily globules was about 2 μm. There were no statistically differences (*P* < .05) in the droplet size over the time of storage.

All results obtained on the lipid emulsion stability after mixing drugs with the PN admixture are summarized in Tables 5 and 6. None of the PN admixtures showed signs of reduced emulsion stability after mixing with any of the drugs in any of the mixed concentrations. Compared with the unmixed controls (Table 4), all physical parameters remained in the same order of magnitude and all were well below the acceptance criteria to be stable and safe.

**Table 5:**
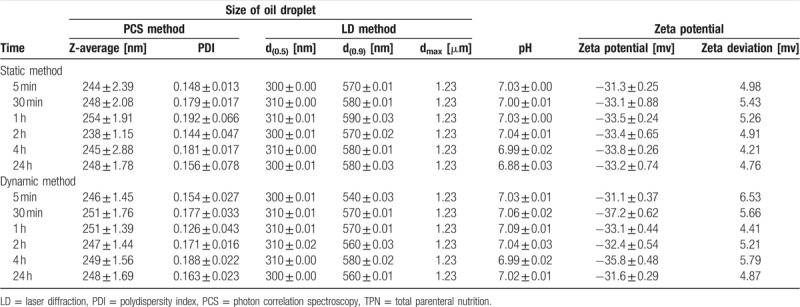
Admixture TPN with furosemide—static and dynamic method.

**Table 6:**
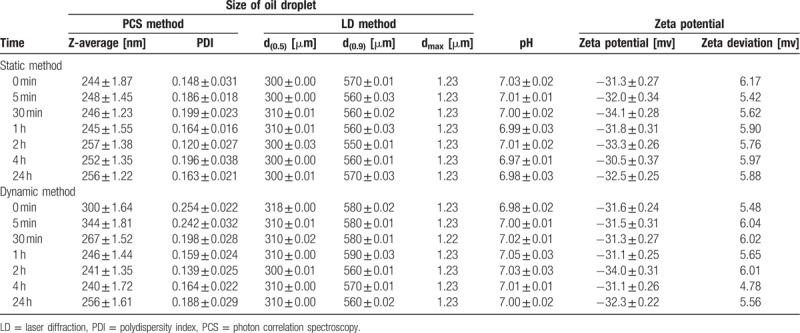
Admixture TPN with torasemide—static and dynamic method.

Using laser diffractometry, the median (d_0.5_) size of oily droplets of the complete TPN admixtures with drugs was 310 ± 10 nm and 90% of oily droplets (d_0.9_) were under 560 ± 20 nm. No oily globules larger than 1.23 μm were detected in any of the admixtures using the laser diffractometry method (Tables 5 and 6). In comparison with the parenteral admixture without the drugs, there were no statistical differences (*P* < .05) in the size of oily droplets.

**Figure 1:**
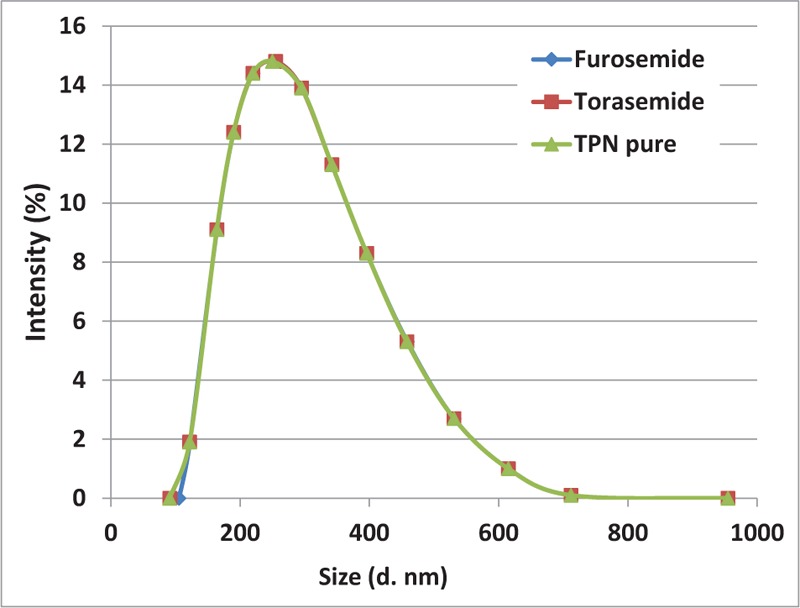
Distribution of lipid droplets of parenteral admixture with furosemide, torasemide, and pure TPN (t = 24 h, dynamic method). TPN = total parenteral nutrition.

Using PCS, the obtained Z-average of the oil droplet size was approximately 250 nm and the index of polydispersity was very narrow (0.160), thereby indicating that all samples were exceptionally monodisperse (Tables 5 and 6). The size of oily droplets was in the same range (*P* < .05), regardless of the storage time with furosemide or torasemide with the TPN admixture (Fig. 1).

The Zeta potential of the parenteral admixture was −30 mV. This parameter of TPN admixture with furosemide as well as with torasemide was in same range, −31 to −35 mV, and did not significantly (*P* < .05) change during the 24-h contact of the drugs with TPN admixtures (Tables 5 and 6).

The pH value in complete TPN admixtures formulated without the drugs was pH 6.50. The addition of both drugs led to an increase in pH, specifically, in the range of 6.97 to 7.09. Compared to t = 0, the pH in drug-containing admixtures did not change (±0.05 of units) during the 24 h of contact with both drugs (Table 6). There were no significant differences (*P* < .05) for both drug types and the contact time.

## 4 Discussion

Dietary protein restriction is a very important factor in nutritional therapy for CKD patients.^[4,5]^ In accordance with the European Society of Parenteral and Enteral Nutrition (ESPEN) Guidelines,^[5]^ 0.6 g of amino acids per kg of body weight was prepared in a new TPN formula. Previous studies showed that low dosages of amino acid are nutritionally safe and provide neutral or slightly positive nitrogen and, concurrently, that patients with advanced stage CKD can fully adapt to low protein regimens.^[5]^ A protein-restricted diet providing not only adequate amounts of amino acids, but also reduced uremic symptoms, delayed the progression of advanced renal disease and dialysis and was associated with prolonged survival.^[4,6]^

The differential buffer composition of nephro solutions is very important because it could prevent worsening of metabolic acidosis, which is a common complication in uremic patients.^[7]^ Metabolic acidosis is related to negative nitrogen balance as well as enhanced protein catabolism. In a chronic condition, it also suppresses albumin synthesis.^[3,5]^ For that reason, we used Aminomel Nephro 6% solutions in our study.

A crucial aspect of nutrition during CKD is the amount of energy supply because during protein restriction, a neutral or positive nitrogen balance requires high energy intake to prevent protein degradation, negative nitrogen balance, and loss of lean body mass.^[6]^ Generally, the energy requirement in nondialyzed, CKD patients should not be different from normal energy expenditure.^[5]^ The composition of MCB is far removed from the protein/energy requirements of CKD patients. It is not possible to provide sufficient energy intake and low dosage of amino acids at the same time with MCB bags.

In this study, 20 nonprotein kcal per kg of body weight was calculated per 0.6 g of amino acids, with 70% of energy derived from glucose and the remaining 30% from lipids. This amount of energy meets the normal energy requirements of HPN patients, but the formulation is unique in comparison with MCB bags which supply significantly higher energy levels. Electrolyte content can be problematic in patients with renal failure, where hypokalemia or hypophosphatemia may occur initially in the course of a disease and can be significantly influenced by diuretic drugs.^[5]^ Electrolyte intake should be determined on an individual basis in patients with renal failure and consequently, the range may be larger. As such, the novel formulations described here were assessed across the full range (Table 2).

Another issue determining the efficacy and safety of HPN in CKD patients is the long-term stability of any proposed PN admixture. Both precipitates and large oil droplets can potentially be dangerous upon infusion, with the potential to lead to pulmonary emboli.^[8]^ The simple light microscope method used in the study is highly sensitive and practical, and it is verified by PCS and the Coulter method. Use of a light microscope facilitates the detection of particles approximately 1 μm in size or enlarged particles up to 100 μm in size. A pure (without drugs) investigated PN admixture was stable up to 8 days at 2°C to 8°C and 24 h at 25°C.

The loop diuretics chosen in this study, furosemide and torasemide, are the most commonly used drugs in CKD HPN patients.^[4]^ Both drugs have the same mechanism of action. Nevertheless, differences in the chemical structure cause these drugs to have different pharmacokinetic properties. Torasemide, in comparison with furosemide, works faster because it has higher bioavailability, it acts longer, has high protein binding, and dosing is less frequent than furosemide because it has a longer half-life.^[9–12]^ Furosemide is used at a dose of 40 to 200 mg daily, dependent upon the response and extracellular fluid volume as well as the frequency determined by specific clinical needs. The equivalent doses of torasemide are from 20 to 100 mg daily. In HPN the effect of oral drugs may be altered due to inadequate gastrointestinal absorption therefore the parenteral administration of diuretics may be required in CKD patient. Where possible, these should be given separately from the TPN solution (intermittently with sufficient rinsing) or adding the medications via a multilumen catheter or via a Y-site connection only if sufficient proof of stability is reported.^[13]^ Intravenous bolus doses of diuretics rapidly increases sodium excretion especially for the first hours and then it progressively declines. Also the peak natriuretic effect with the next doses is less than the first one. Postdiuretic renal sodium and fluid retention are inevitable. This compensatory sodium retention are not related to loop diuretics and to avoid this effects, loop diuretics should be injected at short intervals or infused continuously.^[11]^ The multilumen catheter ensures minimal contact time between the drugs and the TPN solution and hence reduces the risk of incompatibility.^[13]^ Nevertheless, according to ESPEN Guidelines, multilumen catheters are not recommended in HPN patients in order to minimize the risk of infection. Usually tunneled, monolumen, central catheters are used as permanent access in HPN patients.^[14]^ Ideally, the lumen intended for administration of TPN should not be used in addition to further drugs, but this may not be possible in case of HPN patients with CKD. HPN admixture infusion takes usually about 12 h, so simultaneous administration of loop diuretics is required. The Y-site connection has become a necessity, but little information on the compatibility of drugs during Y-site administration with TPN solutions are known.^[13]^ Only the compatibility of furosemide has been tested and previously reported with TPN solutions.^[8,15]^ However, the value of these reports is limited because it cannot be extrapolated to different admixtures composition or drug concentrations. The compatibility of torasemide with PN admixtures has never been performed in any previous study. Diuretics may affect the stability of TPN admixtures as well as TPN admixtures also increasing the risk of unionized drug precipitation. The factor indicating stability is the pH. The pH decreases over time in PN admixtures because of the hydrolysis of fat triglycerides. Additional chemical reactions yielding base or acidic products also affect the pH. For the lipid stability and lecithin emulsifier, a pH range of 5 to 8 is necessary. The negatively charged surface (phosphate moiety) prevents the coalescence of lipid globules. A pH below 5.0 favors lipid instabilities. However, furosemide and torasemide injections are a buffered alkaline solution with a pH of about 9. In our study, there was only a slightly increased pH with a contact reaction with drugs because the TPN admixture possesses a large buffer capacity.^[6]^ On the other hand, at pH values below 7, precipitation of the unionized drugs upon mixing with the less alkaline TPN may occur.^[8]^ The pH of all tested TPN formulations has a value under 7. Therefore, conducting compatibility considerations for newly tailored admixtures for HPN CKD patients and diuretics was essential. The purpose was to generate sufficient data to improve the safety of Y-site administration of loop diuretics with the PN. The major limitation of the present study is that the results are based on physical reactions and the medication concentration was not determined. However, there is a tendency toward a short contact time between solutions administered through a Y-site, so the chemical incompatibility of ingredients is less relevant.^[8]^ Therefore, the present study was designed to simulate Y-site administration in the worst case of administration and check physical incompatibility as an increase in size and/or the number of particles and/or an increase in lipid droplet size, both as compared to the original, nonmixed samples of TPN (Tables 5 and 6). In addition, the results were confirmed statically inspired by the method used by Trissel et al^[15]^ The worst case means that the contact time is the longest and the drug concentration is highest. The calculated rate and usual concentrations of solutions implemented were selected after consultations with physicians and were presented in Tables 1 and 3. Sodium chloride 9 mg/mL was used as drugs diluent. In the presented study, the worst case was connected with Admixture number 1 (the slowest rate of administration—150 mL/h) and furosemide solution in concentration 0.9 mg/mL as well as torasemide in concentration 0.45 mg/mL. The present study confirms some data already published for furosemide^[8,15]^ and provides new data for torasemide and PN because torasemide has not been tested before. Although results for furosemide are similar to a previous study using the TPN admixture with nephro amino acid solution is novel. According to this data, we can predict that all solutions of furosemide up to 0.9 mg/mL and torasemide up to 0.45 mg/mL can be compatible with presented PN composition. However, it should be born in mind that extrapolation of results for another PN composition can be difficult and risky.

## 5 Conclusions

The physical compatibility of 2 loop diuretics, furosemide, as well as torasemide, and a new PN admixture intended for home-treated CKD patients were proved. The increasing concentration of both drugs up to the most extreme mixing ratios estimated between the given drugs and TPN admixtures in the infusion lines were tested. No incompatibilities were found for both diuretics and the proposed composition of the TPN admixture of furosemide and torasemide were compatible with the PN admixture up to 24 h under the applied test conditions. Employing several methods capturing various indicators of incompatibility combined with a range of mixing concentration of tested diuretics ensure efficient and safe therapy. The results are valuable in PN practice for CKD patients. It allows obtaining maximum benefits, minimizing risks, and reducing the progression of renal insufficiency, especially during HPN. It is worth noting that any change of the composition of proposed PN admixture as well as a concentration of the above tested drugs requires new compatibility studies.

## Author contributions

All authors have contributed significantly to the publication. D.W.S. was responsible for conception of the study, contributed to the experimental design, performed the experimental part of the study, and contributed to the writing of the manuscript; M.P. was responsible for the conception and contributed to the experimental design; and S.K. was the supervisor and critically revised the intellectual content of the study. All authors read and approved the final version of the manuscript.

**Conceptualization:** Dorota Watrobska-Swietlikowska.

**Data curation:** Dorota Watrobska-Swietlikowska.

**Formal analysis:** Dorota Watrobska-Swietlikowska.

**Funding acquisition:** Dorota Watrobska-Swietlikowska.

**Investigation:** Dorota Watrobska-Swietlikowska.

**Methodology:** Dorota Watrobska-Swietlikowska, Magdalena Pietka.

**Project administration:** Dorota Watrobska-Swietlikowska.

**Resources:** Dorota Watrobska-Swietlikowska, Magdalena Pietka.

**Software:** Dorota Watrobska-Swietlikowska.

**Supervision:** Dorota Watrobska-Swietlikowska, Stanislaw Klek.

**Validation:** Dorota Watrobska-Swietlikowska.

**Visualization:** Dorota Watrobska-Swietlikowska.

**Writing – original draft:** Dorota Watrobska-Swietlikowska, Magdalena Pietka.

**Writing – review & editing:** Dorota Watrobska-Swietlikowska, Stanislaw Klek, Magdalena Pietka.
